# Effectiveness of the Novel Anti-TB Bedaquiline against Drug-Resistant TB in Africa: A Systematic Review of the Literature

**DOI:** 10.3390/pathogens11060636

**Published:** 2022-06-01

**Authors:** Afsatou Ndama Traoré, Mpumelelo Casper Rikhotso, Ntshuxeko Thelma Banda, Maphepele Sara Mashilo, Jean Pierre Kabue Ngandu, Vuyo Mavumengwana, Andre G. Loxton, Craig Kinnear, Natasha Potgieter, Scott Heysell, Rob Warren

**Affiliations:** 1 Department of Biochemistry and Microbiology, Faculty of Sciences, Engineering & Agriculture, University of Venda, Thohoyandou 0950, South Africa; mputso@yahoo.com (M.C.R.); ntshunxeko@gmail.com (N.T.B.); leratomashilo8@gmail.com (M.S.M.); kabue.ngandu@univen.ac.za (J.P.K.N.); natasha.potgieter@univen.ac.za (N.P.); 2 DSI-NRF Centre of Excellence for Biomedical Tuberculosis Research, South African Medical Research Council Centre for Tuberculosis Research, Division of Molecular Biology and Human Genetics, Faculty of Medicine and Health Sciences, Stellenbosch University, Cape Town 7505, South Africa; vuyom@sun.ac.za (V.M.); gl2@sun.ac.za (A.G.L.); gkin@sun.ac.za (C.K.); rw1@sun.ac.za (R.W.); 3 South African Medical Research Council Genomics Centre, Cape Town 7505, South Africa; 4 Division of Infectious Diseases and International Health, Department of Medicine, University of Virginia, Charlottesville, VA 22903, USA; skh8r@hscmail.mcc.virginia.edu

**Keywords:** bedaquiline (BDQ), drug-resistant TB, treatment, isoniazid (INH), rifampin (RMP), Africa

## Abstract

Background: In 2018, an estimated 10.0 million people contracted tuberculosis (TB), and 1.5 million died from it, including 1.25 million HIV-negative persons and 251,000 HIV-associated TB fatalities. Drug-resistant tuberculosis (DR-TB) is an important contributor to global TB mortality. Multi-drug-resistant TB (MDR-TB) is defined as TB resistant to at least isoniazid (INH) and rifampin (RMP), which are recommended by the WHO as essential drugs for treatment. Objective: To investigate the effectiveness of bedaquiline addition to the treatment of drug-resistant TB infections on the African continent. Methodology: The search engine databases Medline, PubMed, Google Scholar, and Embase were used to obtain published data pertaining to DR-TB between 2012 and 2021 in Africa. Included studies had to document clinical characteristics at treatment initiation and outcomes at the end of treatment (i.e., success, failure, recurrence, loss to follow-up, and death). The included studies were used to conduct a meta-analysis. All data analysis and visualization were performed using the R programming environment. The log risk ratios and sample variances were calculated for DR-TB patients treated with BBQ monotherapy vs. BDQ and other drug therapy. To quantify heterogeneity among the included studies, random effect sizes were calculated. Results: A total of 16 studies in Africa from Mozambique (N = 1 study), Eswatini (N = 1 study), Democratic Republic of the Congo (N = 1 study), South Africa (N = 12 studies), and a multicenter study undertaken across Africa (N = 1 study) were included. In total, 22,368 individuals participated in the research studies. Among the patients, (55.2%; 12,350/22,368) were male while 9723/22,368 (44%) were female. Overall, (9%; 2033/22,368) of patients received BDQ monotherapy, while (88%; 19,630/22,368) patients received bedaquiline combined with other antibiotics. In total, (42%; 9465/22,368) of the patients were successfully treated. About (39%; 8653/22,368) of participants finished their therapy, meanwhile (5%; 1166/22,368) did not finish their therapy, while people (0.4%; 99/22,368) were lost to follow up. A total of (42%; 9265/22,368) patients died. Conclusion: Very few studies on bedaquiline usage in DR-TB in Africa have been published to date. Bedaquiline has been shown to enhance DR-TB results in clinical studies and programmatic settings. Hence, the World Health Organization (WHO) has recommended that it be included in DR-TB regimens. However, in the current study limited improvement to DR-TB treatment results were observed using BDQ on the continent. Better in-country monitoring and reporting, as well as multi-country collaborative cohort studies of DR-TB, can expand the knowledge of bedaquiline usage and clinical impact, as well as the risks and benefits throughout the continent.

## 1. Introduction

Treatment outcomes for drug-resistant tuberculosis (DR-TB) are generally dismal, with low cure rates and high fatality rates [1]. Treatment takes a long time, and many of the medications are not well tolerated [2]. Drug resistance is threatening global TB control, with over 500,000 cases resistant to first-line treatments reported in 2018 [1]. Bedaquiline is a diarylquinoline that inhibits bacterial adenosine triphosphate synthase, a novel antimycobacterial target [3,4]. In 2012, the European Union and the United States granted bedaquiline expedited clearance [5]. It was the first anti-tuberculosis medicine from a novel class in more than 40 years.

In clinical trials and programmatic settings, bedaquiline has improved DR-TB outcomes [5,6,7]. The World Health Organization (WHO) advises it be included in most DR-TB regimens, and many clinical trials of bedaquiline-containing regimens are now underway (WHO, 2019). Despite reclassification of Bedaquiline as a group A medicine for rifampin-resistant and multidrug-resistant (RR/MDR)-TB [5], the WHO promotes field testing of modified regimens for multidrug-resistant (MDR)-TB that use bedaquiline instead of the historically used injectable aminoglycosides or polypeptides.

In Africa, there were 26,845 multidrug-resistant and rifampin-resistant TB (MDR/RR-TB) cases and 867 extensively drug-resistant (XDR)-TB cases reported in 2017 [1]. MDR-TB, defined as TB resistant to at least isoniazid (INH) and rifampin (RMP), which are recommended by the WHO as essential drugs for the treatment of TB and XDR-TB, was recently redefined as MDR-TB plus resistance to any fluoroquinolone (FQ) and at least one of the other group A drugs, bedaquiline or linezolid. Treatment enrolment was extremely low (21% for MDR/RR-TB and 1% for XDR-TB) among all notified multidrug-resistant and rifampin-resistant (MDR/RR)-TB and XDR-TB cases [1]. Subsequently, it is critical to consider how novel medications, such as bedaquiline, should be included in the treatment of DR-TB in Africa as recommended by WHO [5].

Several epidemiological studies on drug resistance, transmission dynamics, and population structure of drug-resistant TB strains have been conducted across Africa [8,9,10,11,12,13]. However, there is a scarcity of systematic data on the use of new drugs in the treatment of MDR-TB in Africa. The objective of this study was to examine the effectiveness of bedaquiline addition to the treatment of drug-resistant TB infections on the African continent. The study also attempted to explore whether adding bedaquiline to the WHO-recommended second-line drug therapy improved patient treatment outcomes.

## 2. Results

### 2.1. Baseline Characteristics of Included Studies

Table 1 summarizes the baseline characteristics of the 16 included studies in the review (Table 1). One (1/16; 6%) study was undertaken in Mozambique, another (1/16; 6%) study in Eswatini, and one (1/16; 6%) study in the Democratic Republic of the Congo. Twelve studies (12/16; 75%) were done in South Africa, with one study (1/16; 6%) being a multicenter study undertaken across 15 countries in Africa [7] (Table 1).

Table 2 summarizes the treatment regimen and treatment outcomes.

The treatment outcomes were categorized into successful, completed, died, treatment failed, and lost to follow-up from the overall patient population of 22,368 who were either on bedaquiline or bedaquiline-containing regimens and those who did not receive any drugs. A total of 2033/22,368 (9%) patients received BDQ monotherapy, while 19,630/22,368 (88%) patients received bedaquiline combined with other antibiotics (Table 2), wherein an overall of 9465/22,368 (42%) were successfully treated with BDQ/BDQ combined therapy. A total of 519/22,368 (2%) patients were not given any antibiotics. From the included studies, about 8653/22,368 (39%) participants finished their therapy; meanwhile 1166/22,368 (5%) did not finish their therapy, while 99/22,368 people (0.4%) were lost to follow-up. A total of 9265/22,368 (42%) died (Table 2).

### 2.2. Meta-Analysis

**Figure 1:**
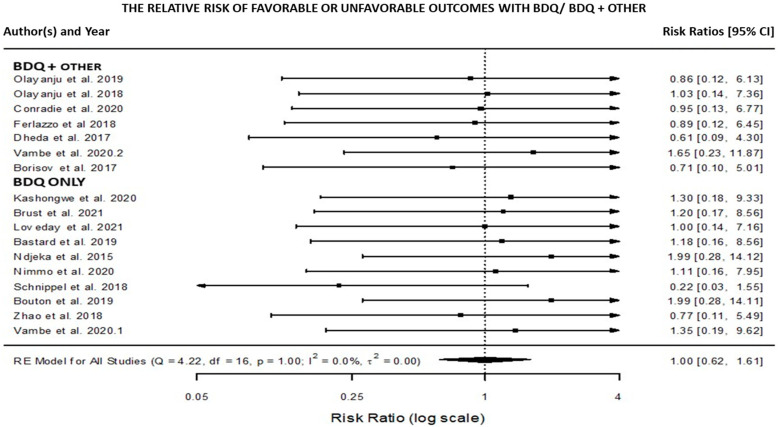
Forest plot with two subgroups (BDQ vs. BDQ + OTHER). The results of the individual studies are shown grouped together according to their subgroup. The summary polygon at the bottom of the plot shows the results from a random-effects model when analyzing all included studies [6,7,8,9,10,11,14,15,17,18,19,20,21,22,23].

The meta-analysis was carried out in order to offer transparent, objective, and reproducible summaries of the study findings (Figure 1). In the statistical analysis of data from cohort, medical, and intervention studies, relative risk/risk ratios are used to determine the strength of the association between treatment exposure and treatment outcomes. The meta-analysis of the included studies assessed the association between DR-TB patients treated with BBQ monotherapy versus BDQ plus other medicines in relation to their treatment outcome (success or failure), as shown in Figure 1.

When compared, DR-TB patients who received BDQ with other drugs had a greater treatment success; DR-TB patients who received BDQ monotherapy had a higher treatment failure as seen in the forest plot Figure 1. Overall, the data imply that while BDQ alone may not support increased therapeutic success in the treatment of DR-TB, BDQ combined with other drugs may increase treatment success in the treatment of DR-TB.

**Figure 2:**
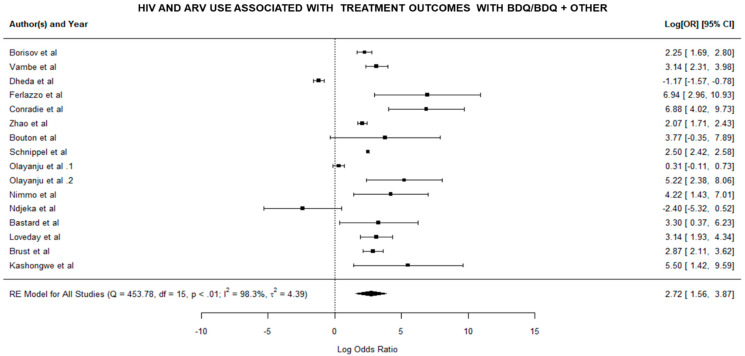
Forest plot showing the positive association between HIV and the use of ARVs in DR-TB therapy with BDQ or BDQ with other drugs. The summary polygon at the bottom of the plot shows the results from a random-effects model when analyzing all included studies [6,7,8,9,11,14,15,17,18,19,20,21,22,23].

To assess the impact of factors that may influence treatment outcomes, a meta-analysis was conducted to assess the impact of HIV infection and the use of antiretroviral medication (ARV) on patients’ treatment success and failure during BDQ therapy. The strength of the association between the occurrences was measured by odds ratios (ORs). There was a link if the OR was greater than one; however, if the OR was less than one, there was no positive association. Figure 2 illustrates the outcomes of the meta-analysis, which revealed heterogeneity among the included studies.

As shown in Figure 2, odds ratios were estimated within the included studies to explore the relationship between HIV patients and ARV usage and treatment outcome (success or failure) using BDQ. When HIV patients and ARV usage were studied, they were shown to be associated with treatment success or failure. The study results showed that HIV and the use of ARVs played a role in the therapeutic effectiveness of DR-TB therapy with BDQ or BDQ in combination with other drugs.

## 3. Discussion

New WHO policy guidelines propose accelerating the use of bedaquiline (BDQ) in shorter and completely oral treatment regimens for multidrug-resistant/rifampicin-resistant TB (MDR/RR-TB) as a replacement for the second-line injectable medication, or as part of a novel shorter regimen under operational research settings [1,5]. They propose that by simplifying the regimen, patients and care teams may be able to better adhere to therapy and achieve cure, as well as enhance programmatic outcomes [24].

Since the introduction of bedaquiline, limited studies in Africa have reported on the use, benefits, and challenges of this drug [25,26]. Hence, the current study aimed to conduct a situational analysis of the introduction and use of the novel drug BDQ in shorter regimens for the treatment of MDR-TB and XDR in patients with TB [8,9,10,11,12,13,21]. To our knowledge, this review contributes to the evaluation and analysis of bedaquiline introduction and usage by healthcare practitioners in Africa, as well as its successes and challenges in the treatment of MDR-TB and XDR-TB patients on the continent.

In the current review, an overall patient population of 22,368 was either on bedaquiline or bedaquiline-containing regimens or did not receive any drugs. A total of 2033/22,368 (9%) patients received BDQ monotherapy, while 19,630/22,368 (88%) patients received bedaquiline combined with other antibiotics (Table 2). In addition, about 519/22,368 (2%) of patients were not given any antibiotics. Overall, a total of 9465/22,368 (42%) were successfully treated, while a total of 9265/22,368 (42%) died (Table 2); meanwhile about 8653/22,368 (39%) participants finished their therapy and 1166/22,368 (5%) did not finish their therapy, while 99/22,368 people (0.4%) were lost to follow-up. The included studies suggested that detection of MDR-TB and the use of BDQ early in the course of infection supports a favorable treatment outcome (successful treatment), as opposed to using BDQ later in the stage of the infection, when resistance may have developed in patients on anti-TB drugs. These findings are in line with other bedaquiline deployment experiences from high HIV and tuberculosis burden areas [27,28,29], which reported that the early use of BDQ resulted in decreases in MDR/RR-TB-related mortality and treatment failure [6]. In addition, the included studies indicated that patients started on bedaquiline had better treatment outcomes [7,9,10,14,23].

The goal of this meta-analysis was to provide transparent, objective, and replicable summaries of the included study findings. Figure 1 illustrates this: only two of the included studies using BDQ monotherapy had favorable outcomes (treatment success), while the other studies had failure outcomes (treatment failure). Only two study studies in the BDQ and other drugs group showed unfavorable outcomes (treatment failure), while the majority of the studies showed favorable outcomes (treatment success) (Figure 1). Overall, the statistical findings revealed that while BDQ alone may not support increased therapeutic success in the treatment of DR-TB, BDQ combined with other drugs might support increased therapeutic success in the treatment of DR-TB. Borisov and colleagues (2017) suggested that the effectiveness of BQD may be overstated due to the additive and/or synergistic effects of other effective drugs (e.g., carbapenems, linezolid, etc.) and that new experimental studies should be conducted to assess the critical effect of bedaquiline, as well as other drugs, in new combination therapies [7]. The included studies were diverse, demonstrating that there was variation among them (Figure 1). In the meta-analysis, however, a strong association was observed (Figure 1).

To assess the impact of HIV and the use of antiretroviral medicine (ARV) on treatment success or failure in patients receiving BDQ therapy or BDQ in combination with other drugs, we calculated the odds ratios between treatment exposure and outcome (Figure 2). The analysis showed positive association between the treatment outcome (success or failure) of individuals taking bedaquiline or bedaquiline combined with other drugs and HIV and ARV usage (Figure 2). The statistical analysis could not quantify the impact of BDQ alone, which is one of the study’s limitations. A study by Olayanju and colleagues (2018) investigated the long-term bedaquiline-related treatment outcomes in patients with extensively drug-resistant tuberculosis and found that few HIV-infected patients had a favorable (successful) treatment outcome after treatment with bedaquiline [18].

The current study findings are similar to a previous study that investigated the incidence and determinants of mortality among people taking second-line anti-tuberculosis drugs in Sub-Saharan Africa. The study found that DR-TB patients with comorbidities had a higher mortality rate [26]. Furthermore, the study suggested that combining modified shorter regimens with newer medications could assist DR-TB patients to achieve better treatment outcomes (treatment success) [26]. The results of the current study revealed that DR-TB patients who took BDQ in combination with other medications had a higher treatment success rate when compared to those who only took BDQ for treatment. Another study indicated that adding bedaquiline to an optimal background regimen was associated with a high rate of favorable (successful) treatment outcomes in patients with DR-TB [4]. A study that investigated the treatment of highly drug-resistant pulmonary tuberculosis found that a combination of bedaquiline, pretomanid, and linezolid led to a favorable (successful) outcome at 6 months after the end of therapy in a high percentage of patients with highly drug-resistant forms of tuberculosis [8]. A study in South Africa looked at the long-term treatment results of patients with severely drug-resistant tuberculosis and found that those who used bedaquiline had a better treatment outcome. Furthermore, bedaquiline had the same effect on HIV-infected patients [18].

There are a number of limitations of this study, including the limited number of published studies on the assessment of bedaquiline in Africa. The included studies did not break down treatment success by MDR, Pre-XD, and XDR groups. Future research is needed to see if adding bedaquiline and the proper use of the drug to the second-line treatment regimen, as recommended by the WHO, improves patient outcomes in Africa. The association of BDQ and BDQ + other combined therapies was assessed, as well as the impact of HIV and ARV usage. However, the impact of BDQ alone could not be confirmed statistically. While it is critical to offer bedaquiline in difficult-to-treat patients, WHO standards should be strictly followed to prevent the development of bedaquiline-resistant TB strains.

## 4. Materials and Methods

### 4.1. Inclusion Criteria

**Figure 3:**
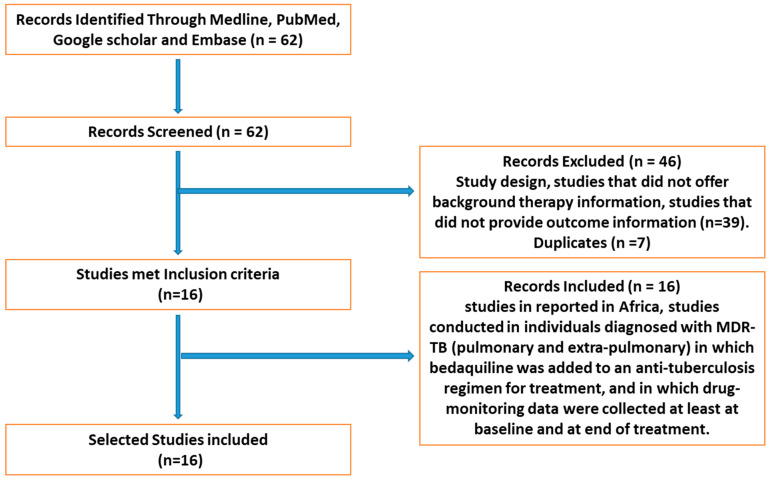
Flow chart of literature search and review process for study selection.

Between 2012 and January 2021, the databases Medline, Pubmed, Google Scholar, and Embase were searched using the following terms: “tuberculosis” + “resistance” + “drug treatment” + “Africa” + “prevalence” + “Bedaquiline.” Full published studies were reviewed and evaluated for eligibility using the following inclusion criteria: (1) studies conducted in Africa; (2) studies conducted in individuals diagnosed with MDR-TB (pulmonary and extra-pulmonary), in which BDQ with or without a control group was included as one of the anti-tuberculosis drug regimens for treatment; (3) studies conducted in which drug-monitoring data (receipt of drug by patients) were collected at least at baseline and at the end of treatment; and (4) studies reported in English (Figure 3). The exclusion criteria were as follows: (1) research that was not carried out in Africa; (2) animal studies; (3) studies that did not offer background therapy information; (4) studies that did not provide outcome information; (5) study duplicates.

A total of 62 studies were found, 46 of which were eliminated due to inclusion requirements. The protocol for this study followed PRISMA guidelines, as previously reported [30]. Table 1 summarizes the 16 studies that satisfied the inclusion criteria. The following variables were extracted from the included studies to generate the current systematic review: author, period, country, population size, age, gender, inpatient/outpatient, HIV status, antiretroviral treatment, patient with MDR/XDR TB, and treatment result of patients.

### 4.2. Statistical Analysis

All data analysis and visualization were performed using the R programming environment version 3.5.0 software (Rstudio, Boston, MA, USA) [31]. A meta-analysis was conducted using the included studies. For statistical analysis, the R packages metafor and meta were utilized. The log risk ratios and sample variances for DR-TB patients treated with BBQ monotherapy vs. BDQ and other drugs therapy were calculated using the escalc function. To quantify heterogeneity among the included studies, random effect sizes were calculated. The odds ratios and random effect sizes of study variables (HIV, ARV) and patients’ treatment outcomes (success, failure) were calculated using the escalc function in R to generate a forest plot of the included studies.

## 5. Conclusions

In conclusion, the goal of this study was to evaluate the introduction and use of bedaquiline, a new anti-TB medicine, in WHO-recommended MDR-TB treatment regimens in Africa. Included studies showed that healthcare practitioners throughout the continent are using BDQ or BDQ-containing regimens to combat drug-resistant TB. The present evaluation, however, found limited improvement to DR-TB treatment results in individuals treated with BDQ or BDQ-containing regimens in the included trials. This might be due to a variety of circumstances, including improper medication management, as suggested by the WHO. The study found a positive association between the treatment outcome (success or failure) of individuals taking bedaquiline or bedaquiline combined with other drugs and HIV and ARV usage. More data throughout the continent are needed, however, to highlight the risks and advantages of using these newer medications to treat MDR-TB. Despite the lack of comprehensive data on the use of new medications in the treatment of MDR-TB in Africa, the information given in this study shows how well the novel anti-TB drug bedaquiline has been received and utilized in WHO-recommended MDR-TB treatment regimens throughout the continent. Future research is needed to determine whether adding bedaquiline to the second-line treatment regimen, as well as the proper use of the drug, as recommended by the WHO, improves patient outcomes in Africa.

## Figures and Tables

**Table 1:** Characteristics of studies containing bedaquiline as an alternative drug for drug-resistant TB treatment.

Author *	Period ***	Country **	Population Size ^#^	Age ^##^	Gender (Male/Female) ^###^	Inpatient ^#^*	Outpatient ^	HIV Status (Positive) ”	On Antiretroviral Therapy ””	Multidrug Resistant/Rifampicin-Resistant **^	Pre-Extensively Drug Resistant ^^^	XDR-TB ^^*
Total	2008–2021	-	22,368	≥15 years	22,073	719	577	15,345	13,670	19,839	220	2124
Borisov et al., 2017 [7]	2008–2016	Africa, Asia, Europe, Oceania and America	428 patients	>15 years	263 Male/165 Female	179	-	94 (22%)	92 (21%)	105 (24%)	-	195 (45%)
Vambe et al., 2020 [10]	2015–2018	Eswatini	352 patients	>18 years	206 Male/145 Female	-	-	271 (77%)	271 (77%)	196 (56%)	46 (13%)	44 (12%)
Dheda et al., 2017 [14]	2008–2012	South Africa	273 patients	-	154 Male/119 Female	-	172	119 (43%)	108 (39%)	-	-	273 (100%)
Ferlazzo et al., 2018 [15]	2016	India and South Africa	28 patients	>18 years	17 Male/11 Female	-	-	11 (39%)	-	2 (7%)	10 (36%)	14 (50%)
Conradie et al., 2020 [8]	2015–2017	South Africa	109 patients	>14 years	57 Male/52 Female	-	-	56 (51%)	-	38 (35%)	-	71 (65%)
Zhao et al., 2019 [16]	2015–2017	South Africa	330 patients	>18 years	190 Male/140 Female	-	-	204 (62%)	94 (28%)	330 (100%)	-	-
Bouton et al., 2019 [17]	2015–2017	South Africa	173 patients	>18 years	91 Male/82 Female	173	-	108 (62%)	-	173 (100%)	-	-
Schnippel et al., 2018 [6]	2014–2016	South Africa	19,617 patients	>15 years	10,959 Male/8658 Female	-	-	13,893 (71%)	12,430 (63%)	18,542 (94%)		1075 (5%)
Olayanju et al., 2018 [18]	2008–2017	South Africa	272 patients	>18 years	161 Male/111 Female	272	-	135 (50%)	125 (46%)	-	-	272 (100%)
Olayanju et al., 2019 [19]	2014–2018	South Africa	63 patients	>18 years	39 Male/24 Female	63	-	37 (59%)	-	-	-	63 (100%)
Nimmo et al., 2020 [9]	2016–2019	South Africa	297 patients	>18 years	45 Male/47 Female	-	297	137 (46%)	297 (100%)	297 (100%)	-	-
Ndjeka et al., 2015 [20]	2013–2014	South Africa	91 patients	>18 years	55 Male/36 Female	-	-	54 (59%)	54 (59%)	-	57 (63%)	34 (37%)
Bastard et al., 2019 [11]	2015–2018	Mozambique	19 patients	>18 years	11 Male/8 Female	-	-	12 (63%)	12 (63%)	19 (100%)	-	-
Loveday et al., 2021 [21]	2013–2017	South Africa	108 patients	-	108 Female	-	108	88 (81%)	74 (68%)	108 (100%)	-	-
Brust et al., 2021 [22]	2016–2018	South Africa	195 patients	>18 years	84 Male/111 Female	-	-	123 (63%)	113 (58%)	29 (15%)	78 (40%)	80 (41%)
Kashongwe et al., 2020 [23]	2016–2017	DR of Congo	32 patients	>18 years	18 Male/14 Female	32	-	3 (9%)	-	-	29 (91%)	3 (9%)

* Author: Author of the published study included. ** County: Country where study was done *** Period: The period when the study was carried out. ^#^ Population size: The number of study participants. ^##^ Age: The age range of participants. ^###^ Gender: The gender of participants. ^#^* Inpatient: Hospitalized patients. ^ Outpatient: Non-hospitalized patients. ” HIV status: Participants who were HIV-positive. ”” On Antiretroviral: Cases of participants taking ARVs. **^ MDR: MDR-TB cases. XDR: ^^* XDR-TB cases and ^^^ Pre-XDR: Pre-XDR-TB cases.

**Table 2:** Included studies’ treatment outcomes.

Author ^^^	BDQ *	Without BDQ **	BDQ + Other Drugs *^	Successful/Cured **^^	Treatment Completion ^#^	Died ^##^	Treatment Failed ^###^	Lost to Follow-Up ”
Total	2033	519	19,630	9465	8653	9265	1166	99
Borisov et al., 2017 [7]	-	-	428/428 (100%)	176/428 (41%)	22/428 (5%)	33/428 (8%)	19/428 (4%)	-
Vambe et al., 2020 [10]	292/352 (83.0%)	-	60 (17%)	139/352 (39%)	1/352 (0.2%)	51/352 (14%)	6/352 (2%)	-
Dheda et al., 2017 [14]	-	-	273/273 (100%)	57/273 (21%)	-	186/273 (68%)	203/273 (74%)	-
Ferlazzo et al., 2018 [15]	-	-	28/28 (100%)	22/28 (78%)	-	1/28 (3%)		1/28 (3%)
Conradie et al., 2020 [8]	-	-	109/109 (100%)	98/109 (90%)	98/109 (90%)	7/109 (6.4%)	11/109 (10%)	1/109 (1%)
Zhao et al., 2019 [16]	162/330 (49%)	168/330 (51%)	-	259/330 (79%)	-	50/330 (15%)	71/330 (21%)	38/330 (11%)
Bouton et al., 2019 [17]	76/173 (43.9%)	97/173 (56.1%)	-	-	-	-	2 (1%)	-
Schnippel et al., 2018 [6]	1016/19,617 (5%)		18,601/19,617 (95%)	8307/19,617 (42%)	8307/19,617 (42%)	8788/19,617 (45%)	763/19,617 (4%)	-
Olayanju et al., 2018 [18]	-	204/272 (75%)	68/272 (25%)	72/272 (26%)	72/272 (26%)	79/272 (29%)	57/272 (21%)	30/272 (11%)
Olayanju et al., 2019 [19]	-	-	63/63 (100%)	45/63 (71%)	45/63 (71%)	-	18/63 (28%)	-
Nimmo et al., 2020 [9]	92/92 (100%)	-	-	73/92 (79.3%)	-	19/92 (20.7%)	-	-
Ndjeka et al., 2015 [20]	91/91 (100%)	-	-	-	58/91 (64%)	3/91 (3%)	5/91 (5.5%)	4/91 (4%)
Bastard et al., 2019 [11]	19/19 (100%)	-	-	13/19 (68%)	3/19 (16%)	-	-	-
Loveday et al., 2021 [21]	58/108 (54%)	50/108 (46%)	-	58/108 (54%)	14/108 (13%)	8/108 (7%)	3/108 (3%)	25/108 (23%)
Brust et al., 2021 [22]	195/195 (100%)	-	-	129/195 (66%)	16/195 (8%)	25/195 (13%)	8/195 (4%)	-
Kashongwe et al., 2020 [23]	32/32 (100%)	-	-	17/32 (53%)	17/32 (53%)	15/32 (46.8%)	-	-

Author ^^^: Author of the published study included. BDQ *: Patients taking BDQ. Without BDQ **: Patients not taking BDQ. BDQ + other drugs *^: Patients taking BDQ and other drugs. Successful/Cured **^^: Successfully treated. Treatment completion ^#^: Completed treatment course. Died ^##^: Died during the study. Treatment failed ^###^: Failed treatment during the study. Lost to follow-up ”: The participants stopped coming for follow-up/checkups.
